# Isolated tuberculosis of the lumbar facet joint: A case report

**DOI:** 10.1097/MD.0000000000028268

**Published:** 2021-12-23

**Authors:** Linan Wang, Zongqiang Yang, Chaoran Wang, Xi Zhu, Jiandang Shi, Ningkui Niu

**Affiliations:** a Clinical Medical College of Ningxia Medical University, Yinchuan, China; b Department of Spine Surgery, General Hospital of Ningxia Medical University, Yinchuan, China.

**Keywords:** facet joint tuberculosis, spinal tuberculosis, surgical treatment

## Abstract

**Rationale:**

Spinal tuberculosis (TB) is the most common in bone and joint TB, of which vertebral TB is more common, while accessory TB is rare. The incidence of isolated adnexal lesions in spinal TB is 2% to 3%. It is difficult to distinguish the imaging changes of spinal adnexal TB from other types of spinal infections and spinal tumors, and the pathological diagnosis of spinal TB is often atypical. Here, we report a case of isolated lumbar facet joint TB.

**Patient concerns:**

A 64-year-old female patient had an 8-month history of low back pain, decreased pinprick sensation in the left anterior middle thigh area, weakening of the patellar tendon reflex of the left lower limb, and enhanced MRI of the lumbar vertebrae showed bone destruction at the left superior and inferior articular process of the lumbar 2 to 3 and the encapsulated calcification containing the lesion around the articular process. The enhanced scan showed solid part and septal enhancement, and the lesion protruded to the left and posterior side of the spinal canal, and the left posterior edge of the dural sac was compressed at the same level. Conservative treatment for 8 months was ineffective.

**Diagnoses:**

L2–3 vertebral lamina, facet joint, and intraspinal space-occupying Lamina TB.

**Interventions:**

The diagnostic treatment scheme for anti-TB drugs was routinely administered before the operation. Isoniazid (300 mg), rifampicin (450 mg), ethambutol (750 mg), and pyrazinamide (1500 mg) were administered orally once daily after breakfast for 1 month, as anti-TB treatment for 1 month. Posterior lumbar total laminectomy and decompression, pedicle screw internal fixation, TB focus debridement, lumbar intertransverse process bone graft fusion was performed 1 month later.

**Outcomes:**

The patient was relieved of symptoms after surgical treatment and anti-tubercular medication.

**Lessons:**

We present a case of isolated TB of the lumbar facet joint, which was initially diagnosed as L2–3 vertebral lamina, facet joint, and intraspinal space-occupying osteochondroma. For patients with long-term low back pain, it is suggested to follow-up with lumbar computed tomography and lumbar magnetic resonance imaging when conventional X-ray examination does not show any lesion. Despite its rarity, isolated TB of the lumbar facet joint should be highly suspected in elderly patients with pulmonary TB, low-grade fever, and waist pain.

## 1 Introduction

Spinal tuberculosis (TB) is the most common form of bone and joint TB, of which vertebral TB is more common, while accessory TB is rare, accounting for only 3% of all spinal TB.^[1,2]^ The structures of the posterior accessory of the spine are not clearly displayed on plain X-ray film, often causing delayed diagnosis. Meanwhile, it is difficult to distinguish the imaging changes of spinal adnexal TB from other types of spinal infections and spinal tumors, and the pathological diagnosis of spinal TB is often atypical.^[3]^ Therefore, spinal facet joint TB often leads to misdiagnosis in clinical work, and there are usually many complications in the late stage. Accessory TB combined with bone destruction usually causes spinal instability or neurological dysfunction, and can also cause progressive scoliosis.^[4–6]^ The incidence of isolated adnexal lesions in spinal TB is 2% to 3%,^[1,7]^ while isolated lumbar facet joint TB is rarely reported.

## 2 Case report

### 2.1 General information

The patient, a 64-year-old female farmer, was admitted to the hospital on July 14, 2020, mainly because of “low back pain for 8 months, aggravated for 1 month.’. Before 8 months, there was no obvious inducement of lumbar and back pain. There was a sense of flatulence, aggravated after activity, and slightly relieved after rest. Also present were limited waist activity, conscious nocturnal pain, numbness in the front of the left thigh, no lower limb pain, normal defecation, and defecation function. Oral nonsteroidal drug treatment slightly improved low back pain, but the symptoms worsened in nearly 1 month, for which the patient went to an orthopedic clinic. During the course of the disease, there was no low fever, fatigue, night sweats, and no weight loss. The contact history of TB was ruled out, and there was no family history of tumor.

### 2.2 Physical examination

The patient had a normal gait, straightened spine, extension, flexion, scoliosis, and limited rotation. The spinous process and paraspinal tenderness and percussion pain of the lumbar vertebrae 2 and 3 were obvious. The pinprick sensation of the left anterior middle thigh area was decreased, the muscle strength of the iliopsoas and quadriceps of the left lower limb was grade 5, the knee-tendon reflex of the left lower limb was weakened, the muscle tension of the left lower limb was normal, and the muscle strength and muscle tension of the right lower limb were normal. The right knee-tendon reflex and Achilles tendon reflex were normal. Pathological signs were not observed. The left straight leg raising test was 45 °, and the right straight leg raising test was 85°. The sellar region was normal and the anal sphincter was not relaxed (Table 1). The visual analog scale (VAS) and Oswestry Disability Index (ODI) were used to evaluate lumbar pain and lumbar function.^[8]^ The preoperative VAS score was 8, and the ODI score was 66.6.

**Table 1:** Results of specialist physical examination.

	Left	Right		Left	Right
Hip flexors	5	5	Patellar clonus	Negative	Negative
Knee extensors	5	5	Ankle clonus	Negative	Negative
Ankle dorsiflexors	5	5	Babinski's sign	Negative	Negative
Long toe extensors	5	5	Chaddock's sign	Negative	Negative
Ankle plantar flexors	5	5	Oppenheim's sign	Negative	Negative
Muscular tension	Normal		Gordon's sign	Negative	Negative
Voluntary anal contraction	Yes	Yes	Lasegue sign	Positive	Negative
Patellar tendon reflex	+	++	Bragard sign	Positive	Negative
Achilles tendon reflex	++	++			

Pin-prick sensation of left anterior middle thigh area is decreased; Pin-prick sensation of right extremities and saddle area is normal.

### 2.3 Laboratory examination

Preoperative blood routine findings: white blood cell count: 6.05∗10^9^/L, neutrophil count: 2.46∗10^9^/L, neutrophil ratio: 58.4%, lymphocyte count: 0.90∗109/L, lymphocyte ratio: 36.2%, hemoglobin: 98.0 g/L, red blood cell count: 4.33∗10^12^/L; erythrocyte sedimentation rate (ESR): 2.20 mg/L; C-reactive protein (CRP): 12 mm/h; alkaline phosphatase: 60 U/L. T cells spot test of TB infection (TSPOT.TB) suggested the following: Antigen A: 3 SFCs/2.5∗10∼5PBMCs, Antigen B: 108 SFCs/2.5∗10∼5PBMCs; Standard Tube Agglutination Test: Negative; Tumor markers: CA19-9, CA12-5, and CEA were normal.

### 2.4 Imaging examinations

**Figure 1:**
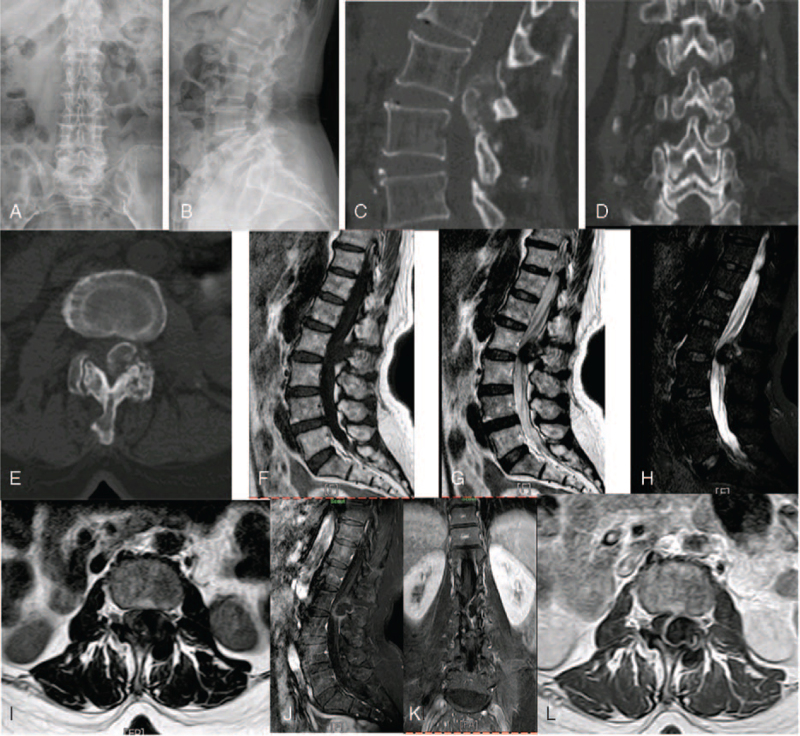
Preoperative images: (A, B) Lumbar anteroposterior and lateral radiographs: mass like calcification shadow can be seen at the left facet joint of lumbar 2–3; (C—E) CT plain scan of lumbar vertebrae: hyperosteogeny at the edge of lumbar vertebrae, bone destruction of the left superior and inferior articular process of lumbar vertebrae at lumbar 2 and 3 levels and slightly high density around the vertebral body, the focus protruded into the spinal canal, the size was about 2.7 x 1.8 x 4.0 cm, the shape was irregular, the edge was calcified, the internal density was uneven and multiple dotted high-density shadows were seen; (F—L) MRI enhancement of lumbar vertebrae: irregular cystic solid masses were seen outside the spinal canal on the left side of lumbar 2 and 3 vertebrae, especially solid components, bone destruction of the superior and inferior articular process and encapsulated calcification around articular process were seen. The enhanced scan showed solid part and septal enhancement, compression of the adjacent spinal cord, and the lesion grew laterally through the left intervertebral foramen of lumbar 2–3 and 3–4.

Digital radiography showed a mass-like calcification shadow at the left facet joint of waist 2 to 3 (Fig. 1A,B). Computed tomography revealed bone destruction at the left adnexa of the lumbar 2 to 3 vertebrae and local soft-tissue masses (Fig. 1C--E). Enhanced MRI of the lumbar vertebrae showed bone destruction at the left superior and inferior articular processes of the lumbar 2 to 3 and encapsulated calcification-containing lesion around the articular process. The enhanced scan showed solid part and septal enhancement, and the lesion protruded to the left and posterior side of the spinal canal, and the left posterior edge of the dural sac was compressed at the same level (Fig. 1F--I). Chest radiography and CT showed lobular consolidation in the lingual segment of the upper lobe of the left lung (considered an old lesion) and scattered small nodules were present in the middle and upper lobes of the right lung; no obvious abnormality was found on EMG and SEP.

Diagnosis: L2–3 vertebral lamina, facet joint, and intraspinal space-occupying lamina TB.

### 2.5 Surgical treatment

The diagnostic treatment scheme for anti-TB drugs was routinely administered before the operation. Isoniazid (300 mg), rifampicin (450 mg), ethambutol (750 mg), and pyrazinamide (1500 mg) were administered orally once daily after breakfast for 1 month, as anti-TB treatment for 1 month. However, the symptoms of the patient were not significantly improved, and the patient had lower limb neurological dysfunction.

**Figure 2:**
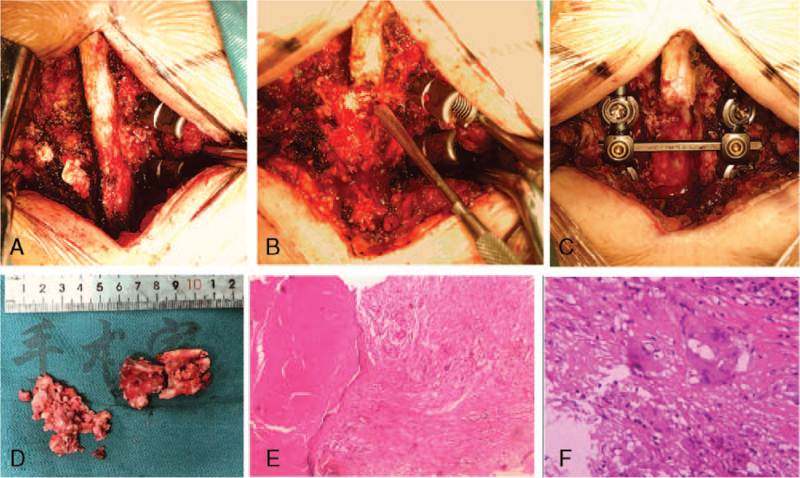
(A, B) General photos during operation: A large amount of cheese-like necrosis can be seen in the left lamina and facet joint; (C) After complete laminectomy, the lesion was completely removed, and the left L2 and L3 nerve roots and dural sac were completely released; (D) Photographs of gross pathology specimens taken during surgery; (E,F) The pathological examination of the specimens performed after surgery: (E, HE staining × 100), (F, HE staining × 400) Bone and necrotic tissue, fibrous tissue between the trabecular bone in some areas showed proliferation, lymphocytes, epithelioid cells, and multinucleated giant cell reactions were seen around the necrotic lesions in some areas.

Total lumbar laminectomy and decompression, posterior lumbar focus debridement, posterior lumbar pedicle screw fixation, and lumbar intertransverse process bone graft fusion were performed under combined intravenous and inhalation anesthesia (CIIA) on July 17, 2020. Surgical findings: The spinous process, lamina, articular process, and transverse process of L2–3 were exposed, and a large amount of caseous necrosis was observed in the left vertebral lamina and facet joint considered as TB necrotic material (Fig. 2A--C) and diagnosed as TB based on frozen section. The insertion points of the bilateral L2 and L3 pedicle screws were located at the midpoint of the transverse process, and 2 sets of pedicle screws were inserted. Intraoperative exploration revealed bone destruction of the left L2 and L3 pedicles as well as breakage of the medial wall. The whole lamina of L2–3 was completely removed by the ultrasonic bone knife, intraoperatively, complete destruction of the facet joint in the left L2 and L3 was found, with massive caseous tissue formation, which protruded into the spinal canal and occupied approximately 80% of the volume of the spinal canal, compressing the dural sac and deforming the nerve root in the left L2 and L3. Intraoperative exploration of the L2–3 discs showed no abnormalities. After complete laminectomy, the lesion was completely removed, and the left L2 and L3 nerve roots and dural sac were completely released. After alternating irrigation with iodophor and normal saline, there was no active bleeding or macroscopic lesion tissues. A gelatin sponge was used to cover the dural sac, and a pair of transverse links was placed. The resected spinous process bone was cut into small pieces and mixed with allogeneic bone, and then implanted into the right facet joint and bilateral transverse process of L2–3. Two negative pressure drainage tubes were placed and fixed, and the incision was closed. After the operation, the tissue samples obtained during surgery (Fig. 2D) were sent to pathology, general bacterial culture, and Gene Xpert MTB/RIF detection (X-pert).

Postoperative pathological diagnosis showed bone and necrotic tissue and fibrous tissue between the trabecular bone; some areas showed proliferation, lymphocytes, epithelioid cells; multinucleated giant cell reactions were observed around the necrotic lesions in some areas and were more likely to be TB (Fig. 2E,F). Xpert MIB-RIF rpoB and mutation detection of *Mycobacterium tuberculosis* indicated wild-type *M tuberculosis*. General bacterial culture and identification showed aseptic growth.

### 2.6 Postoperative follow-up

**Figure 3:**
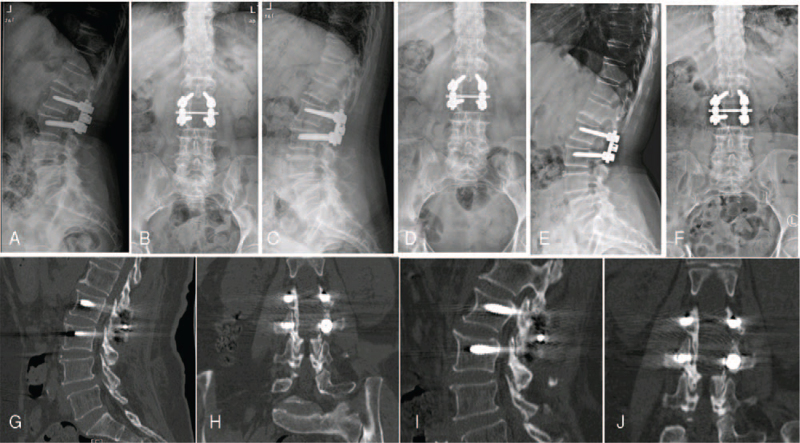
(A, B) were the anteroposterior and lateral radiographs of the lumbar spine on the 1 month after the operation, respectively, showed the internal fixation was in good position. Three months (C, D, G, H) and half a year (eE, F, I, J) after the operation, reexamination of lumbar anteroposterior and lateral radiographs and lumbar CT showed that the internal fixation device was in good position, and partial fusion of facet joint could be seen.

After the operation, the patient reported that the pain in the back and numbness in the left lower limb were significantly relieved compared with before, and the VAS score was 4 points on the first postoperative day. Oral quadruple anti-TB medication was continued and administered regularly after surgery: isoniazid (300 mg QD), rifampicin (450 mg QD), ethambutol (750 mg QD), and pyrazinamide (1500 mg QD). One month after the operation, the anteroposterior and lateral radiographs of the lumbar vertebrae showed that the internal fixation device was in good position (Fig. 3A,B); the VAS score was 2 points. Reexamination of blood routine showed white blood cell count: 5.04∗10^9^/L, neutrophil count: 2.21∗10^9^/L, neutrophil ratio: 55.3%, lymphocyte count: 0.84∗10^9^/L, lymphocyte ratio: 35.3%; ESR: 3.10 mg/L; CRP: 9 mm/h. Three months after the operation (Fig. 3C,D,G,H) and half a year later (Fig. 3E,F,I,J), reexamination of lumbar anteroposterior and lateral radiographs and lumbar CT showed that the internal fixation device was in a good position, and partial fusion of the facet joint could be seen. Routine blood tests, ESR, and CRP were normal 3 and 6 months after the operation, and the VAS and ODI scores were 1, 42.3, 0, and 28.8, respectively.

## 3 Discussion

Spinal TB is usually transmitted by the blood and is caused by *M tuberculosis*. Vertebral marginal TB, also known as epiphyseal type, is the most common type of vertebral TB, accounting for approximately 75% of TB cases in the spine, and posterior structures of the spine are relatively rare. The posterior vertebral venous plexus runs on the surface of the vertebral body and reaches around the spinous process, transverse process, and articular process.^[9]^*M tuberculosis* can reach the posterior structure of the spine through the venous plexus, resulting in infection of the posterior structure of the spine.^[10]^ The most common infection site of posterior spinal TB is the pedicle, with a prevalence rate ranging from 0.2% to 10%. When spinal TB involves the posterior structure, it is often accompanied by anterior disc and vertebral body lesions, but there are few reports on the incidence of isolated facet TB without the anterior column of the spine. Narlawar et al^[1]^ found that among 1076 patients with spinal TB, 19 (1.76%) had lesions in the anterior column of the spine that involved the posterior facet joint at the same time. Ashwin et al^[5]^ reported a 14-year-old male patient with spinal TB, who was diagnosed with isolated L4–5 right facet joint TB, accompanied by L5-S1 isthmic spondylolisthesis and left mild scoliosis. Regular anti-TB drug treatment and lumbosacral orthosis support were administered for 2 months after TB was diagnosed by puncture biopsy, but the patient had progressive worsening of pain with increasing coronal spinal instability. Therefore, L4-S1 pedicle internal fixation and transverse interprocedural fusion were performed via the Wiltse approach, and the patient's symptoms were completely relieved after the operation. Chadha et al^[11]^ reported 3 patients with spondylolisthesis combined with spinal TB at the same level and found that spondylolisthesis may be caused by pressure overload on posterior structures, which may be caused by severe infection of posterior structures, or by severe destruction of anterior structures. However, no patients had infection of isolated posterior structures.

The isolated TB of the lumbar 2 to 3 facet joint reported in this case is often misdiagnosed as a tumor, and the posterior structures of the spine are not clear on radiography, which often leads to missed diagnosis and delayed treatment. The patient had normal preoperative inflammatory indicators, atypical imaging examination, low back pain, and progressive aggravation of symptoms of nerve compression in the left lower extremity. Despite the regular use of anti-TB drugs during the course of the disease,^[4]^ the inflammatory symptoms around the lesions may be significantly improved, but spinal deformities and instability would still exist or worsen progressively. Compared with the most common vertebral marginal TB, the spinal deformity and bone loss caused by facet joint TB are out of proportion, suggesting that unilateral isolated facet joint TB is more likely to cause coronal spinal instability.^[5]^ Therefore, lesion removal, nerve decompression, and spinal stabilization should be used to treat lumbar facet TB. In this case, the patient was treated with short segment fixation and fusion, laminectomy, and decompression, which achieved the purpose of lesion clearance while stabilizing the spine, with satisfactory clinical outcomes.

## 4 Conclusion

For patients with lumbar facet joint TB, in addition to effective anti-TB drug treatment, surgery is indicated when spinal instability occurs and the tuberculous lesion invades the spinal canal and compresses the spinal cord or nerve. Treatment involves early posterior pedicle screw fixation, TB lesion debridement, and lumbar intertransverse process bone graft fusion, which can cure lumbar facet joint TB and maintain the stability of the spine.

## Author contributions

All authors have made substantial contributions to the conception of this study. All authors approved the final manuscript as submitted and agree to be accountable for all aspects of the work.

**Conceptualization:** Linan Wang.

**Data curation:** Chaoran Wang, Xi Zhu.

**Writing – original draft:** Linan Wang, Zongqiang Yang.

**Writing – review & editing:** Jiandang Shi, Ningkui Niu.
